# Draft Genome Sequence of Streptococcus anginosus UMB7768, Isolated from a Woman with Recurrent UTI Symptoms

**DOI:** 10.1128/MRA.00418-20

**Published:** 2020-05-21

**Authors:** Sarah Miller, Taylor Miller-Ensminger, Adelina Voukadinova, Alan J. Wolfe, Catherine Putonti

**Affiliations:** a Bioinformatics Program, Loyola University Chicago, Chicago, Illinois, USA; b Department of Microbiology and Immunology, Stritch School of Medicine, Loyola University Chicago, Maywood, Illinois, USA; c Department of Biology, Loyola University Chicago, Chicago, Illinois, USA; d Department of Computer Science, Loyola University Chicago, Chicago, Illinois, USA; University of Maryland School of Medicine

## Abstract

Streptococcus anginosus recently was implicated as a pathogen involved in urinary tract infections. A strain of *S. anginosus* was isolated from the female urogenital tract. Here, we present the draft genome sequence of *S. anginosus* strain UMB7768.

## ANNOUNCEMENT

Streptococcus anginosus is a catalase-negative Gram-positive bacterium normally associated with a beneficial role in the human gastrointestinal and oropharynx microbiota (1, 2). However, recent studies have shown that *S. anginosus* is a pathogenic contributor to several abscess-forming infections and potentially plays a role in cystic fibrosis (3, 4). Furthermore, *S. anginosus* is the predominant member of the S. milleri group (which also includes S. constellatus and S. intermedius) found in the urogenital tract (5). A prior study found that strains of the bladder and vaginal communities were highly similar, suggesting microbial sharing between these two microbiota (6). *S. anginosus* is considered an emerging uropathogen; it has been associated with urgency urinary incontinence (7) and urinary tract infections (UTIs) (8, 9). Here, we present the draft genome sequence of *S. anginosus* UMB7768 from the urinary microbiota of a female with recurrent UTI.

A voided urine specimen was collected from a patient at the Women’s Pelvic Medicine Center at the University of California, San Diego, as part of a previous institutional review board (IRB)-approved study (University of California, San Diego, IRB no. 170077AW). *S. anginosus* UMB7768 was isolated from this sample using the expanded quantitative urinary culture (EQUC) protocol (10). The genus and species of this isolate were determined with matrix-assisted laser desorption ionization–time of flight (MALDI-TOF) mass spectrometry (10) and then stored at −80°C. Following freezing, the isolate was streaked onto a Columbia nalidixic acid (CNA) agar plate and incubated at 35°C with 5% CO_2_ for 24 h. A single colony was selected from the plate and inoculated in blood heart infusion (BHI) broth at 37°C with shaking for 24 h. DNA was extracted using the Qiagen DNeasy blood and tissue kit following the manufacturer’s protocol for Gram-positive bacteria (with minor modification) and quantified using a Qubit fluorometer. The extracted DNA was sent to the Microbial Genomic Sequencing Center (MiGS) at the University of Pittsburgh for sequencing. There, the DNA was enzymatically fragmented using an Illumina tagmentation enzyme, and then indices were attached using PCR. The DNA library was sequenced using an Illumina NextSeq 550 platform, producing 1,513,634 pairs of 150-bp reads. Raw reads were trimmed using Sickle v1.33 (https://github.com/najoshi/sickle) and assembled using SPAdes v3.13 with the “only assembler” option for k values of 55, 77, 99, and 127 (11). The genome coverage was calculated using BBMap v38.47 (https://sourceforge.net/projects/bbmap/). PATRIC v3.6.3 (12) was used to annotate the genome in-house; the publicly available genome sequence was annotated using the NCBI Prokaryotic Genome Annotation Pipeline (PGAP) v4.11 (13). Unless otherwise noted, default parameters were used for all software tools listed.

The *S. anginosus* UMB7768 draft genome sequence is 2,158,792 bp long, assembled in 47 contigs with an *N*_50_ value of 121,537 bp and a GC content of 38.77%. The genome’s coverage is 182×. The PGAP annotation found 2,168 coding DNA sequences (CDSs), 2,000 with protein prediction, 45 tRNAs, and 5 complete rRNA sequences (3 5S, 1 16S, and 1 23S). PATRIC identified numerous virulence factors and genes associated with antibiotic resistance. Further investigation of the latter using the ResFinder v3.2 (14) Web server identified resistances to macrolides and tetracycline, which have been documented as common within the *S. milleri* group (4). Future examination of the virulence factors identified will expand our understanding of *S. anginosus* in the urogenital tract and its role in UTI development.

### Data availability.

This whole-genome shotgun project has been deposited in GenBank under the accession no. JAAUWB000000000. The version described in this paper is the first version, JAAUWB010000000. The raw sequencing reads have been deposited in the SRA under the accession no. SRR11441032.
